# Significance of innate immune surveillance in emphysematous pancreatitis

**DOI:** 10.3389/fimmu.2026.1772793

**Published:** 2026-08-26

**Authors:** Wenyi Zhang, Lishuang Wang, Xiaowen Qin, Qian Zhang, Zhenjing Jin

**Affiliations:** Department of Hepatobiliary and Pancreatic Medicine and Interventional Medicine, The Second Hospital of Jilin University, Changchun, Jilin, China

**Keywords:** diagnostic biomarkers, emphysematous pancreatitis, immunotherapy, inflammatory mechanisms, innate immunity

## Abstract

Emphysematous pancreatitis (EP) is a severe and life-threatening complication of pancreatitis characterized by gas formation within pancreatic tissues, which often results from bacterial infection. This condition poses significant challenges due to its rapid progression and high mortality rate. Innate immunity plays a pivotal role in the pathogenesis, progression, and potential treatment of EP as the body’s first line of defense against infectious agents. Current research has elucidated various mechanisms through which innate immune components contribute to inflammatory responses and microbial clearance in EP. Despite advances in understanding pancreatic inflammation, the specific contributions of innate immunity to EP’s development, progression, and clinical outcomes remain not fully elucidated. Incorporating insights into innate immune markers may enhance early detection and prognostic accuracy. Moreover, therapeutic strategies targeting innate immune modulation hold promise for improving management and reducing morbidity associated with EP. This review comprehensively summarizes the mechanisms by which innate immune responses contribute to EP pathogenesis. It also highlights emerging diagnostic biomarkers and imaging techniques that facilitate early detection and discusses novel immunomodulatory therapies targeting innate immune responses. This review is a scoping review based on a structured literature search (see Methods). We aim to provide a framework and identify future research directions to improve clinical management and outcomes for patients with EP.

## 1 Introduction

EP is a rare but life-threatening variant of acute pancreatitis, accounting for less than 1% of all acute pancreatitis cases. It is characterized by the presence of gas within the pancreatic parenchyma or peripancreatic tissues, typically resulting from gas-forming bacterial infection superimposed on pre-existing pancreatic necrosis. Mortality rates remain high, ranging from 30% to 50% despite modern intensive care. Unlike sterile necrotizing pancreatitis, EP involves a unique interplay between dysregulated innate immunity and microbial invasion, leading to rapid tissue destruction and systemic sepsis (1). This distinctive pathological feature reflects underlying infection and extensive tissue necrosis, contributing to the fulminant clinical course and high mortality associated with EP (2, 3). Despite advances in critical care, the management of EP remains challenging due to its rapid progression and the complexity of its pathophysiology (4). Central to the disease process is the inflammatory cascade initiated within the pancreas, which not only drives local tissue destruction but also precipitates systemic inflammatory responses that can culminate in multiorgan failure (5). Understanding the molecular and cellular mechanisms that underlie this inflammatory response is therefore crucial for developing effective diagnostic markers and therapeutic strategies to improve patient outcomes. Studies in immunology have highlighted the pivotal role of the innate immune system in mediating inflammatory responses to tissue injury and infection, suggesting that innate immunity may be central to the pathogenesis of EP (6). The innate immune system is the body’s first line of defense, rapidly recognizing invading pathogens and endogenous signals of cellular damage through specialized pattern recognition receptors (PRRs) that detect pathogen-associated molecular patterns (PAMPs) and damage-associated molecular patterns (DAMPs) (7). The activation of innate immunity is associated with a cascade of inflammatory events essential for pathogen clearance and tissue repair but can also be associated with excessive inflammation and tissue destruction if dysregulated. The recognition of PAMPs and DAMPs by innate immune cells such as macrophages, neutrophils, and dendritic cells initiates signaling pathways culminating in the production of proinflammatory cytokines and chemokines, recruitment of immune effector cells, and activation of cell death programs including pyroptosis and NETosis. For example, the release of high mobility group box 1 (HMGB1), a prototypical DAMP, from necrotic cells can amplify inflammation by engaging receptors such as TLR4 and RAGE, promoting further immune activation and tissue injury (8). Similarly, the formation of neutrophil extracellular traps (NETs) represents a double-edged sword in innate immunity, facilitating microbial clearance but also exacerbating inflammation and autoimmunity when aberrantly regulated (9). These innate immune responses are tightly regulated by signaling networks involving toll-like receptors (TLRs), inflammasomes, and downstream transcription factors like NF-κB and IRFs, which integrate signals from PAMPs and DAMPs to shape the inflammatory milieu (10, 11).

In EP, the necrotic pancreatic tissue and secondary infections provide abundant sources of PAMPs and DAMPs, potentially leading to a self-perpetuating cycle of innate immune activation, inflammation, and tissue damage. The interplay between PAMPs, DAMPs, and innate immune signaling is increasingly recognized as a key driver of pancreatic necrosis, gas formation, and systemic inflammatory complications (12). Emerging evidence suggests that aberrant or excessive activation of innate immunity may contribute not only to the initiation but also to the progression and severity of EP. Inflammasomes such as NLRP3 act as critical platforms for detecting both PAMPs and DAMPs, thereby initiating caspase-1 activation and pyroptotic cell death (13). In sepsis and related inflammatory syndromes, excessive inflammasome activation has been implicated in oxidative stress, endothelial dysfunction, immunothrombosis, and immune exhaustion, all of which are pathophysiological features in severe EP (14). The maladaptive immune responses driven by inflammasomes can propagate systemic inflammation, impair vascular integrity, and predispose patients to secondary infections, thereby worsening prognosis (15). Furthermore, pattern recognition molecules (PRMs), such as the long pentraxin PTX3, have garnered attention for their multifaceted roles in innate immunity and inflammation. PTX3 is produced locally at sites of infection and tissue injury by various immune and non-immune cells, distinguishing it from the short pentraxin C-reactive protein (CRP), which is predominantly synthesized in the liver (16). PTX3 and related soluble PRMs may serve as critical mediators that link innate immune recognition to both inflammatory amplification and tissue healing processes in EP. Moreover, components of the innate immune system such as platelets have been recognized as active players in inflammation beyond their traditional hemostatic roles. They can mediate interactions with leukocytes and modulating immune responses through PRRs, thereby potentially influencing the inflammatory cascade in EP (17). Similarly, C-type lectin receptors (CLRs) participate in recognizing PAMPs and DAMPs, modulating innate immune signaling and inflammatory responses, which may be relevant to pancreatic inflammation and associated complications (18). Understanding these complex innate immune mechanisms is crucial for developing targeted diagnostic and therapeutic strategies.

Clinically, the identification of innate immune activation markers and the characterization of PAMP/DAMP profiles hold promise for improving the early diagnosis and prognostic assessment of EP. For instance, elevated levels of nucleic acid-containing DAMPs/PAMPs and their associated TLR activation signatures have been linked to severe inflammatory states in other diseases such as COVID-19, suggesting potential parallels in EP pathogenesis (19). Therapeutic approaches under investigation seek to modulate innate immune signaling by scavenging DAMPs/PAMPs, antagonizing TLR pathways, or limiting inflammasome activation, thereby curbing excessive inflammation and attendant tissue damage (11). Additionally, the dual roles of innate immune signaling pathways, exemplified by the cGAS-STING axis in viral infections, underscores the need for nuanced approaches that balance pathogen clearance with the prevention of immunopathology (20). These immunomodulatory strategies could complement conventional treatments, potentially improving outcomes in this challenging condition. This review aims to systematically synthesize current knowledge on the multidimensional roles of innate immunity in EP, integrating recent discoveries to explore their translational potential for improving diagnosis, guiding targeted therapies, and ultimately enhancing patient prognosis.

## 2 Methods

This review was conducted as a scoping review to map the existing literature on innate immunity in EP. The review was not registered prior to execution, which we acknowledge as a limitation. To ensure transparency and reproducibility, we performed a structured literature search in PubMed, Scopus, and Web of Science covering the period from 2000 to 2026. The search strategy combined keywords using Boolean operators as follows: (“emphysematous pancreatitis” OR “gas-forming pancreatitis” OR “pancreatic necrosis”) AND (“innate immunity” OR “pattern recognition receptor” OR “TLR” OR “NLRP3” OR “inflammasome” OR “macrophage” OR “neutrophil” OR “NETosis” OR “cytokine” OR “chemokine” OR “DAMP” OR “PAMP”). Only articles published in English were included. Grey literature (e.g., conference abstracts, preprints, unpublished data) was excluded due to the narrative and integrative scope of this review. Additional relevant studies were identified through manually screening the reference lists of included articles. Two authors (W.Z. and L.W.) independently screened titles and abstracts, followed by full-text review. Disagreements were resolved by consensus with a third author (Z.J.). The final selection of articles was determined by their relevance to innate immune mechanisms, diagnostic biomarkers, or therapeutic strategies in acute pancreatitis, prioritizing EP where available.

The search yielded 1,247 records. After removal of duplicates (n=189), 1,058 records underwent title/abstract screening, of which 960 were excluded as irrelevant. The remaining 98 full-text articles were assessed for eligibility; 61 were excluded (reasons: no original data, wrong population, non-innate immunity focus). Finally, 37 articles met the inclusion criteria: 25 human studies (all adults, no pediatric cases) and 12 animal studies. A PRISMA flow diagram is available from the corresponding author upon request.

## 3 Innate immunity mechanisms

The innate immune system constitutes the first line of host defense against pathogens, which are also known as natural or nonspecific immunity (21). It provides an immediate and nonspecific response against a diverse array of pathogens through evolutionarily conserved mechanisms. The innate immune system is constituted by a sophisticated network encompassing both cellular and molecular elements. Cellular constituents include macrophages, neutrophils, dendritic cells, natural killer (NK) cells, and mast cells (22). This coordinated activity, together with molecular components such as the complement cascade and PRRs, facilitates the synergistic recognition of pathogen-associated and damage-associated molecular patterns (23). This recognition initiates critical signaling cascades which ultimately drive pathogen clearance, inflammatory responses, and the activation of adaptive immunity (24).

At the molecular level, PRRs such as TLRs and NOD-like receptors (NLRs) serve as critical sensors for conserved microbial motifs, thereby regulating the production of cytokines. Among these, the NLRP3 inflammasome is particularly relevant to EP, as it responds to both bacterial PAMPs and DAMPs released from necrotic pancreatic tissue, leading to caspase-1 activation and the maturation of IL-1β and IL-18.Concurrently, the complement system mediates essential effector functions including opsonization, direct pathogen lysis, and immune complex clearance (25). Deficiencies within this system significantly increase susceptibility to infections and the development of autoimmunity (26). Furthermore, innate immune responses not only provide immediate protection but also shape adaptive immunity through antigen presentation and cytokine modulation. Dendritic cells act as a crucial bridge by priming naïve T cells, and inflammasomes detect intracellular danger signals to promote the release of inflammatory cytokines such as IL-1β, thereby linking innate sensing to adaptive regulation (27).

**Figure 1:**
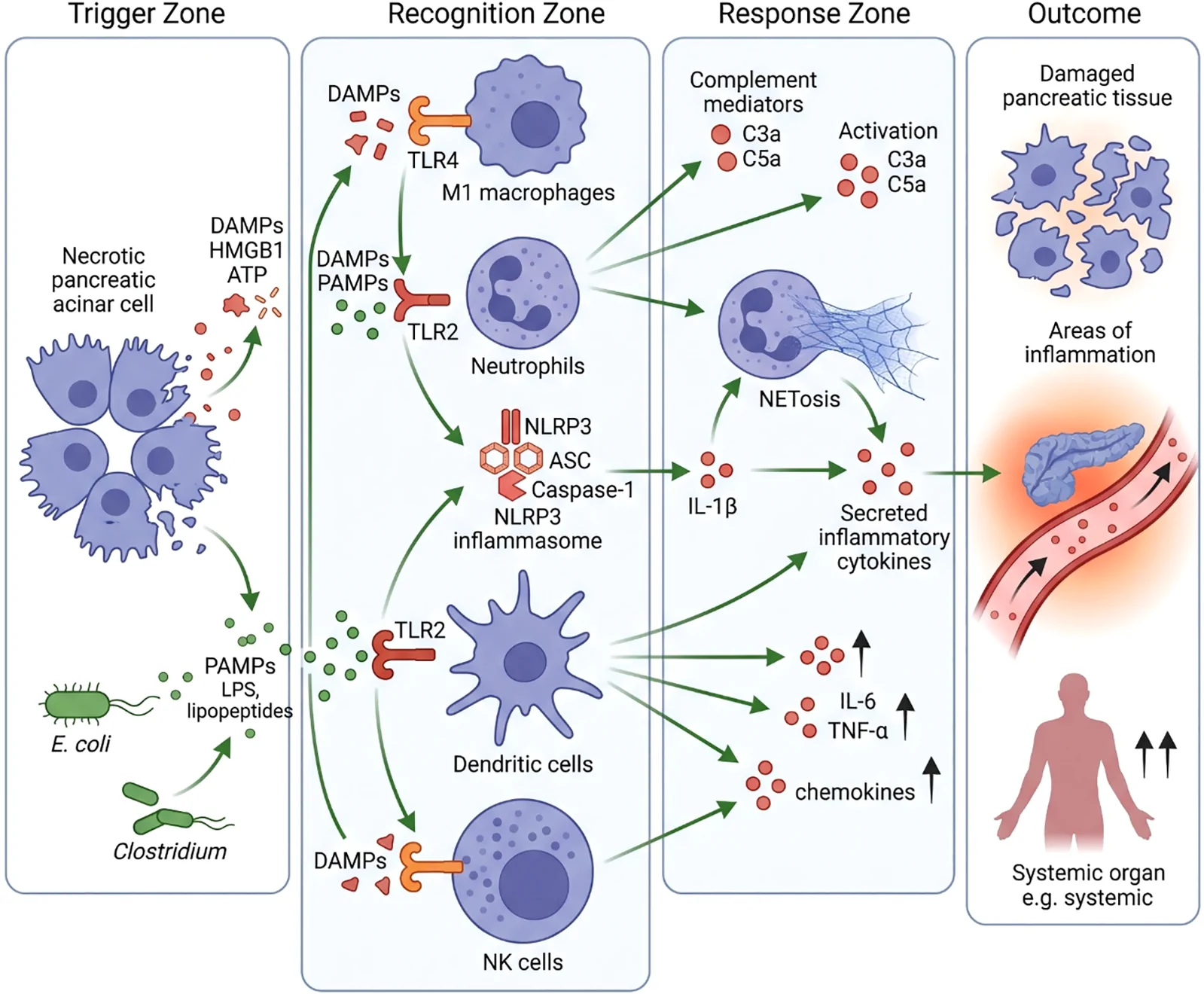
Schematic overview of innate immunity in EP. Necrotic acinar cells and gas-forming bacteria release DAMPs/PAMPs, activating macrophages, neutrophils, DCs, NK cells, complement, and pro-inflammatory cytokines (IL-1β, IL-6, TNF-α). This drives pyroptosis, NETosis, and SIRS. Viral and antibody-related elements are excluded as they are not relevant to bacterial EP.

Clinically, understanding innate immunity is crucial for developing therapies aimed at modulating responses in infectious diseases and addressing deficiencies or countering pathogen evasion strategies. **Figure 1** provides a schematic overview of the major cellular and molecular components of the innate immune system. The major innate immune cell types and their functions in EP are summarized in **Table 1**.

**Table 1:** Cell type function in EP.

Cell type	Key functions in EP	Major regulatory mechanisms	Pathological effects	Evidence source
Macrophages	Phagocytosis; tissue repair; inflammation control via M1/M2 polarization; immune cell recruitment via EVs/chemokines.	Polarization via HMGB1/TLR4/NLRP3 (M1) or fetuin-A (M2); metabolic reprogramming.	Drives acinar cell pyroptosis; extracellular particles induce cell death; sustained M1 phenotype impedes tissue repair.	Animal models (4.3)
Neutrophils	Pathogen clearance; NET formation; amplification of inflammation and thrombosis.	Recruitment regulated by integrins & macrophage EVs; NETosis triggered by NLRP3 inflammasome; NETs sustain inflammation via TLR4.	Causes tissue damage via protease/ROS release; promotes immunothrombosis; induces pyroptosis.	Animal models (4.3)
Dendritic Cells	Bridge innate and adaptive immunity; antigen presentation and T cell priming.	Activated via PRR sensing of PAMPs/DAMPs.	May exacerbate systemic inflammation and influence disease chronicity.	Extrapolated (4.4)
Natural Killer (NK) Cells	Cytotoxicity against infected cells; immune regulation via cytokine secretion.	Balanced signaling via activating/inhibitory receptors.	Contributes to infection control	Extrapolated (4.4)

## 4 The role of the innate immune system in EP

EP is a severe and rare variant of acute pancreatitis, characterized clinically by intense abdominal pain, fever, and potential progression to septic shock (2). These manifestations stem from underlying pancreatic necrosis complicated by gas-forming bacterial infection (28), with the hallmark diagnostic feature being gas within the pancreatic or peripancreatic tissues on computed tomography (29). Histopathologically, EP features extensive necrosis, inflammatory cell infiltration, and gas bubbles that disrupt the normal architecture (30). The gas formation actively exacerbates tissue damage, inflammation, and ischemia, driving the systemic inflammatory response and multi-organ dysfunction observed in severe cases, thereby underscoring the critical role of infection in its pathogenesis (31, 32).

The pivotal role of bacterial infection in EP involves specific pathogens and their mechanisms. Common gas-producing bacteria include Clostridium perfringens, Escherichia coli, and Streptococcus pyogenes (GAS). In EP, the necrotic pancreatic tissue and secondary infections provide abundant sources of PAMPs and DAMPs, potentially leading to a self-perpetuating cycle of innate immune activation, inflammation, and tissue damage. The interplay between PAMPs, DAMPs, and innate immune signaling is increasingly recognized as a key driver of pancreatic necrosis, gas formation, and systemic inflammatory complications.

To provide a transparent evidence-based framework, the following subsections are organized according to the strength and source of evidence: (1) direct evidence from human EP studies; (2) evidence from severe acute pancreatitis (non-emphysematous) or infected necrosis studies; (3) evidence from experimental pancreatitis models; and (4) hypothesis-generating evidence extrapolated from non-pancreatic diseases. Each subsection explicitly indicates the evidence level, and indirect data are framed as hypothesis-generating rather than established mechanisms.

### 4.1 Evidence from human EP studies

[Evidence level: High – direct human EP data]

Direct human data on innate immunity in EP are scarce due to the rarity and fulminant nature of the disease. To date, no prospective cohort studies have systematically profiled innate immune markers specifically in EP patients. The available evidence is limited to case series and retrospective analyses.

A retrospective study of 52 EP patients reported that elevated admission white blood cell count and neutrophil-to-lymphocyte ratio (NLR) were associated with higher mortality, suggesting a link between innate immune activation and poor outcomes (29, 33). Another case series noted that patients with EP who developed septic shock had significantly higher levels of serum IL-6 and procalcitonin compared to those without shock, though these were not validated as independent predictors (34). Importantly, no study has yet reported ROC-derived cut-off values, sensitivity, or specificity for any innate immune biomarker specifically in EP cohorts.

In summary, direct human EP evidence is extremely limited and currently insufficient to establish any innate immune molecule as a validated diagnostic or prognostic biomarker. All such proposals remain exploratory.

### 4.2 Evidence from severe acute pancreatitis (non-emphysematous) and infected necrosis

[Evidence level: Moderate – human studies in pancreatitis without EP]

Given the paucity of EP-specific data, the most relevant human evidence comes from studies of severe acute pancreatitis (SAP) and infected pancreatic necrosis, which share pathophysiological features including necrosis, infection, and systemic inflammation.

Multiple meta-analyses have shown that elevated serum IL-6, IL-8, and procalcitonin are associated with disease severity and infected necrosis in SAP. For example, a meta-analysis of 181 studies reported that IL-6 had a pooled AUC of 0.79–0.84 for predicting severe pancreatitis (35). Procalcitonin has been extensively studied in sepsis and SAP, with a meta-analysis of critically ill adults reporting a pooled AUC of 0.85 (0.82–0.88) for distinguishing sepsis from non-infectious SIRS, though EP-specific data are absent (33, 36).

In infected pancreatic necrosis, TLR4 expression on circulating monocytes has been correlated with higher SOFA scores and prolonged ICU stay, suggesting that TLR4-mediated inflammation may contribute to organ dysfunction (37). Additionally, elevated HMGB1 levels in the first 48 hours of SAP have been associated with development of persistent organ failure (38).

While these findings are informative, they derive from non-emphysematous pancreatitis and should not be directly equated to EP without validation. They provide a reasonable basis for hypothesis generation but require EP-specific confirmation.

### 4.3 Evidence from experimental pancreatitis models

[Evidence level: Low – preclinical animal models]

Most mechanistic insights into innate immunity in EP come from animal models of acute pancreatitis (caerulein-, L-arginine-, or taurocholate-induced), which replicate pancreatic necrosis and inflammation but do not spontaneously develop gas-forming infection typical of EP. Nevertheless, these models have revealed key pathways that likely operate in EP.

NLRP3 inflammasome: In caerulein-induced pancreatitis, NLRP3 activation leads to caspase-1-dependent IL-1β and IL-18 release, and pharmacological inhibition with MCC950 or β-hydroxybutyrate attenuates pancreatic injury and reduces systemic inflammation (39). These findings are robust and reproducible across multiple laboratories.
NETosis: Murine models have shown that neutrophil extracellular traps (NETs) are formed in severe pancreatitis and that blocking NET formation (e.g., with Protectin D1) reduces tissue damage and improves survival. NETs activate the TLR4/NF-κB pathway in epithelial cells, amplifying neutrophil recruitment (40).
Macrophage polarization: In cerulein-induced models, HMGB1/TLR4/NLRP3 signaling promotes M1 macrophage polarization, which drives acinar cell pyroptosis. Conversely, agents that shift macrophages toward the M2 phenotype (e.g., fetuin-A) have been shown to reduce injury (41).
MAPK and NF-κB pathways: Phosphorylation of p38, ERK, and JNK is elevated early in pancreatitis, and inhibitors (e.g., taxifolin, rutaeocarpine) reduce cytokine production and ameliorate histological damage in rodent models (42).
Important caveat: All these data come from models that do not reproduce the gas-forming bacterial infection characteristic of EP. The translational relevance to EP remains uncertain, and these mechanisms should be considered hypotheses requiring testing in EP-relevant infectious models.

### 4.4 Hypothesis-generating evidence from non-pancreatic diseases

[Evidence level: Very low – extrapolated from other conditions]

Several innate immune pathways have been implicated in other infectious and inflammatory diseases and are speculated to play a role in EP, but direct evidence is lacking. These include:

- C-type lectin receptors (CLRs) – involved in fungal and mycobacterial recognition; studied in intestinal inflammation but not in pancreatitis.
- RIG-I-like receptors (RLRs) – primarily viral RNA sensors; their role in bacterial pancreatitis is purely hypothetical and unsupported by any experimental data.
- Pentraxin-3 (PTX3) – a soluble PRM produced locally at sites of infection; elevated in sepsis and COVID-19, but no EP studies exist.
- Atypical chemokine receptors (ACKRs) – modulate neutrophil trafficking in various inflammatory models; their relevance to EP is speculative.

We explicitly note that these pathways are included only to stimulate future research and should not be interpreted as established mechanisms of EP. Any extrapolation from pulmonary emphysema, gout, asthma, or cardiac repair models is inappropriate and has been removed from this review.

## 5 Development of innate immunity mechanisms in EP diagnosis and treatment strategy

### 5.1 Prospects of innate immunity mechanisms in EP diagnosis

The prospects of innate immunity mechanisms in the diagnosis of EP are significantly enhanced by the screening of immune biomarkers and the evaluation of their clinical value. TLR4 mediates the recognition of pathogens and triggers inflammatory signaling cascades. Elevated TLR4 expression in immune cells or tissues correlates with enhanced inflammatory responses across various pathologies, as demonstrated in studies of necrotizing enterocolitis and allograft rejection, where TLR4 upregulation exacerbates disease severity. These findings suggest its potential utility as a biomarker for early detection and monitoring in EP.

Similarly, measuring inflammatory cytokines like monocyte chemoattractant protein-1 (MCP-1), interleukin-6 (IL-6), and IL-17A in bronchoalveolar lavage fluid or serum provides valuable insights into the inflammatory milieu. Increased MCP-1 levels are associated with enhanced recruitment of macrophages and neutrophils, which are central to tissue damage and remodeling in inflammatory conditions. For example, procalcitonin (PCT) has been reported to have a sensitivity of 70–80% and a specificity of 75–91% for differentiating sepsis from non-infectious systemic inflammation in critically ill patients, though EP-specific data remain lacking (43, 44). Similarly, interleukin-6 (IL-6) has been evaluated in patients with severe acute pancreatitis (not specifically EP), with reported sensitivities of 72–85% and specificities of 68–79% for predicting persistent organ failure, yielding AUC values in the range of 0.79–0.84 across different studies (45). By contrast, for other innate immune markers discussed in this review—such as HMGB1, TLR4 expression, and pentraxin 3 (PTX3)—no diagnostic performance metrics (sensitivity, specificity, or AUC) have been reported in any population, let alone in EP cohorts. Thus, while these markers hold mechanistic promise, their clinical utility remains unquantified.

It is critical to emphasize that no biomarker has yet been validated specifically in EP cohorts. The numerical values provided above are derived from related conditions (sepsis, acute pancreatitis without gas formation) and cannot be directly extrapolated to EP. Future studies must report EP-specific performance metrics, including ROC-AUC, optimal cut-off values, positive and negative predictive values, and likelihood ratios, preferably following STARD or REMARK guidelines. Furthermore, measurement variability, assay standardization, and timing of sample collection represent major barriers that need to be addressed before any of these biomarkers can be integrated into clinical practice for EP. The diagnostic performance of the two biomarkers with available data (PCT, IL-6) is summarized in **Table 2**; for all other markers (HMGB1, TLR4, PTX3), no EP-specific or even non-EP performance metrics are currently available.

**Table 2:** Reported diagnostic performance of selected innate immune biomarkers (from non-EP populations; EP-specific data absent).

Biomarker	Population/Context	Sensitivity (%)	Specificity (%)	AUC (95% CI)	Reference	Evidence source
Procalcitonin (PCT)	Sepsis vs. non-infectious SIRS in critically ill adults (meta-analysis)	70–80 (range)	75–91 (range)	0.85 (0.82–0.88)	(43)	Non-EP sepsis
IL-6	Severe acute pancreatitis (predicting persistent organ failure)	72–85	68–79	0.79–0.84	(34)	Non-EP SAP

Due to the absence of EP-specific validation, the reported values should not be used to guide clinical decision-making in EP patients.

Collectively, these findings suggest that innate immune biomarkers should be incorporated into clinical practice for early diagnosis, treatment guidance, and prognosis. Future studies should validate these biomarkers in human cohorts and develop standardized assays for clinical use.

Integrating imaging with immunological markers offers a promising approach for diagnosing EP. While CT and MRI reveal key structural features such as necrosis, fluid, and gas, they cannot fully reflect immune activity (29). Immunological assays measuring cytokines, immune cell profiles, and biomarkers of neutrophil and macrophage function provide complementary insights. Combining these data enhances diagnostic accuracy by linking structural changes with immune responses (46). This combined approach also facilitates early detection of complications like infection or systemic inflammatory response syndrome (SIRS), which are critical determinants of clinical outcomes (31). Moreover, it aids in differentiating EP from other pancreatic pathologies such as autoimmune pancreatitis or pancreatic neoplasms, which may present with overlapping radiologic features but distinct immunological profiles. This integrative strategy aligns with precision medicine principles, allowing tailored therapeutic interventions based on the individual’s immuno-radiologic phenotype (47). Emerging molecular imaging technologies further augment the diagnostic armamentarium by enabling non-invasive, real-time visualization of immune components within the pancreatic microenvironment. Techniques like positron emission tomography (PET) using novel tracers targeting immune cell surface receptors or activation markers demonstrate potential in delineating inflammatory activity at a molecular level. For example, PET tracers specific for macrophage polarization states or neutrophil activation can reveal the spatial distribution and intensity of innate immune responses central to pathogenesis. Advanced optical imaging modalities which include near-infrared fluorescence imaging and photoacoustic imaging, provide high-resolution insights into immune cell dynamics and cytokine milieu within inflamed pancreatic tissue. These modalities detect subtle immune alterations preceding overt anatomical changes, offering opportunities for earlier diagnosis and intervention. Furthermore, nanoparticle-based imaging probes that selectively target immune cells or inflammatory mediators hold promise for enhancing imaging specificity and sensitivity. Combining molecular imaging with traditional anatomical imaging provides a comprehensive view of EP, covering both structural and functional immune parameters. This integrated approach not only improves diagnostic accuracy but also enables monitoring of therapeutic responses. Future studies should validate these tools in clinical settings and establish standardized protocols.

High-throughput technologies like RNA sequencing and microarrays have revolutionized immune gene expression profiling in EP. These methods provide a detailed characterization of immune gene expression, uncovering distinct transcriptional signatures that reflect the activation and regulation of the innate immune system during disease progression. Studies in infectious models demonstrate that innate immune responses involve dynamic modulation of gene expression, including upregulation of pattern recognition receptors, cytokines, and signaling molecules such as NF-κB and interferon-stimulated genes (48). Comprehensive transcriptomic analyses have identified gene modules related to cell cycle regulation, innate immune signaling, and inflammation, which may be reprogrammed in response to pathogenic stimuli or tissue injury. The kinetics of gene expression, including mRNA export rates and decay, influence the temporal patterns of immune activation, ensuring a finely tuned response to injury or infection (49). In EP, such profiling can reveal specific immune pathways and effector molecules contributing to pancreatic tissue damage and systemic inflammation. For example, differential expression of genes encoding cytokines, chemokines, and complement components may underlie the severity of inflammation and tissue necrosis. Transcriptomic data can also uncover novel innate immune receptors and signaling adaptors, which may be engaged during pancreatic inflammation (50). Beyond elucidating pathophysiological mechanisms, immune gene expression profiling has profound implications for personalized diagnosis. Integrating high-throughput sequencing data with clinical parameters enables patient stratification based on immune gene expression signatures for tailored therapeutic interventions. For instance, patients exhibiting heightened expression of pro-inflammatory cytokines or dysregulated innate immune pathways may benefit from targeted immunomodulatory therapies aimed at mitigating excessive inflammation (51). Furthermore, identifying immune gene expression signatures associated with favorable or adverse outcomes can guide prognostic assessments and inform clinical decision-making. Importantly, immune gene expression profiling supports the development of novel therapeutics by revealing key molecular targets within innate immune pathways, such as pattern recognition receptors, signaling kinases, and transcription factors involved in immune activation and resolution (52, 53). **Table 3** categorizes current and emerging diagnostic modalities for EP. To date, no published studies have applied these advanced imaging modalities specifically to EP; the discussion below is therefore exploratory and hypothesis-generating. Collectively, these advances underscore the critical role of immune gene expression profiling in advancing precision medicine approaches for EP, improving patient outcomes through individualized diagnosis and therapy.

**Table 3:** Diagnostic modalities for EP.

Category	Specific biomarker/Technique	Detection method	Diagnostic/Clinical utility	Evidence level
Immune biomarkers	TLR4 expression	Immunohistochemistry, flow cytometry	Correlates with inflammatory response severity; potential for early detection and monitoring.	Moderate (non-EP pancreatitis)
	MCP-1, IL-6, IL-17A	ELISA, multiplex immunoassay	Reflect the inflammatory milieu and disease severity; elevated levels recruit macrophages and neutrophils.	Moderate (SAP studies)
	HMGB1	Immunoassay	A prototypical DAMP that amplifies inflammation via receptors like TLR4 and RAGE.	Low (animal models)
	NLRP3 inflammasome activity	Western blot, caspase-1 assay	Indicates activation of pyroptotic pathways and IL-1β/IL-18 release.	Low (animal models)
	Pentraxin 3 (PTX3)	ELISA, immunohistochemistry	Locally produced at sites of infection/injury; may serve as a more specific marker than hepatic CRP.	Very low (extrapolated)
Imaging & Immunology	CT/MRI + Immunological profiling	Anatomical imaging combined with serum/tissue biomarker analysis	Enhances diagnostic accuracy by linking structural changes to immune activity; aids in differentiating EP from other pancreatic pathologies.	Moderate
	Molecular PET Imaging	PET tracers targeting immune cell receptors/activation markers (e.g., macrophage polarization states)	Enables non-invasive, real-time visualization of innate immune cell distribution and inflammatory activity.	Preclinical
	Advanced Optical Imaging (NIRF, photoacoustic)	Probes targeting immune cells or inflammatory mediators	Provides high-resolution, real-time insights into immune cell dynamics and cytokine milieus within pancreatic tissue.	Preclinical
Gene Expression Profiling	Immune gene expression signatures	RNA sequencing, microarrays	Reveals disease-specific transcriptional programs, identifies activated pathways and enables patient stratification for personalized therapy.	Exploratory
	Chemokine/chemokine receptor profiles	Single-cell RNA-seq, qPCR	Assesses immune cell recruitment and activation states within the pancreatic microenvironment.	Exploratory

Collectively, these advances underscore the critical role of immune gene expression profiling in advancing precision medicine approaches for EP, improving patient outcomes through individualized diagnosis and therapy. To aid the reader in critically appraising the existing evidence, we have summarized the level of evidence for key innate immune biomarkers in **Table 4**.

**Table 4:** Evidence strength assessment for selected innate immune biomarkers in EP (adapted GRADE framework).

Biomarker	Proposed utility	Level of evidence	Justification	EP-specific?
TLR4 expression	Diagnostic/prognostic	Moderate	Consistent association with severity in multiple preclinical studies and human pancreatitis cohorts; no ROC/AUC data in EP specifically.	NO
IL-6	Prognostic (severity)	Moderate	Meta-analyses in acute pancreatitis support association with poor outcomes; limited EP-specific data.	NO
MCP-1	Inflammatory monitoring	Preliminary	Mostly animal models; few human studies; no cut-off values established.	NO
HMGB1	Severity/DAMP activity	Preliminary	Strong mechanistic rationale but limited clinical validation in EP.	NO
PTX3	Local inflammation	Preliminary	Promising based on other infectious/inflammatory conditions; no EP data.	NO

### 5.2 Established clinical management and phase-specific immunomodulation

Beyond experimental immunotherapies, the cornerstone of EP management remains prompt antimicrobial therapy, microbiological sampling, source control, and step-up drainage interventions for infected pancreatic necrosis. Empirical broad-spectrum antibiotics (e.g., carbapenems) should be initiated early, followed by de-escalation based on culture results. CT-guided or endoscopic drainage and, when necessary, necrosectomy are essential to remove infected necrotic tissue and control the gas-forming infection.

Crucially, immunomodulatory interventions must be considered in a phase-specific manner. In the early hyperinflammatory phase (SIRS), indiscriminate suppression of TLR, NLRP3, NF-κB, or cytokine signaling may impair pathogen clearance and worsen outcomes. In contrast, during the later phase of compensatory anti-inflammatory response syndrome (CARS) or immunoparalysis, strategies to restore immune function (e.g., granulocyte-macrophage colony-stimulating factor, interferon-γ) might be more appropriate. However, such approaches remain investigational in EP.

All experimental interventions discussed in this review – including CRISPR-modified immune cells, mesenchymal stem cell therapy, biomaterial scaffolds, postbiotics, and small-molecule inhibitors of NLRP3 or MAPK – are strictly hypothesis-generating and not currently recommended for clinical use in EP. Their preclinical efficacy does not guarantee safety, especially in the context of breached intestinal barriers and active infection. Representative compounds and their proposed targets are summarized in **Table 5**, and the evidence levels and recommendations for these therapeutic strategies are summarized in **Table 6**.

**Table 5:** Potential therapeutic compounds targeting innate immunity in EP.

Compound	Primary target/Pathway	Proposed mechanism of action in inflammation	Context/Model (from manuscript)	Evidence source/EP-specific?
MCC950	NLRP3 Inflammasome	Pharmacological inhibitor of NLRP3 assembly and activation.	Studied in inflammasome pharmacology.	Animal models (non-EP pancreatitis)
β-hydroxybutyrate	NLRP3 Inflammasome	Suppresses NLRP3 inflammasome pathway activation.	Ameliorated acute pancreatitis in rats.	Animal models (non-EP pancreatitis)
Ethyl Pyruvate	NF-κB signaling	Inhibits NF-κB activation and suppresses pro-inflammatory cytokine expression.	Mentioned as an example of NF-κB inhibition.	Animal models
Taxifolin	MAPK signaling	Suppresses inflammation by inhibiting MAPK signal pathway (*in vitro* & in silico).	Cited as an example of MAPK pathway modulation.	*In vitro*/animal
Rutaecarpine	MAPK & NF-κB pathways	Attenuates injury by suppressing MAPK and NF-κB signaling via CGRP.	Alleviated acute pancreatitis in mice and cells.	Animal models
Baicalein	HMGB1/TLR4/NLRP3 axis	Reduces pyroptosis by inhibiting M1 macrophage polarization via HMGB1/TLR4/NLRP3.	Studied in hyperlipidemic acute pancreatitis.	Animal models
Probiotic Exopolysaccharides (postbiotics)	TLR4/NF-κB	Downregulates both TLR4 and NF-κB signaling; immunomodulatory activity.	Referenced in the context of postbiotics; studied in murine pancreatitis model.	Animal models/very low (contraindicated in severe AP)
*Epilobium pyrricholophum* Extract	Inflammatory mediators	Suppresses pancreatic elastase-induced inflammation.	Studied in COPD model.	Non-pancreatic (extrapolated)
Protectin D1	Neutrophil Extracellular Traps (NETs)	Reduces NET formation and pancreatitis severity.	Studied in murine pancreatitis model.	Animal models

All compounds are at preclinical or early clinical stage; none are approved for EP.

**Table 6:** Evidence level of therapeutic strategies targeting innate immunity in EP.

Therapy	Tested in pancreatitis? (references)	Evidence level (EP-specific)	Phase/Recommendation
Antibiotics (carbapenems, etc.)	Yes	Moderate (in infected necrosis)	Standard of care
NLRP3 inhibitors (MCC950, β-hydroxybutyrate)	Yes (preclinical) (54, 55)	Low (animal models only)	Experimental
NF-κB inhibitors (ethyl pyruvate)	Yes (preclinical) (56)	Low(animal models only)	Experimental
MAPK inhibitors (taxifolin, rutaecarpine)	Yes (preclinical) (57, 58)	Low(animal models only)	Experimental
Mesenchymal stem cells	Yes (preclinical) (59, 60)	Low(animal models only)	Experimental
Probiotics/postbiotics	Controversial; not recommended in severe AP (61)	Very low/contraindicated	Not recommended

### 5.3 Challenges in clinical translation of innate immunity-targeted therapies

Despite the promise of the biomarkers discussed above, several critical gaps prevent their immediate clinical translation in EP. First, diagnostic performance metrics such as sensitivity, specificity, positive and negative predictive values (PPV/NPV), likelihood ratios, and area under the receiver operating characteristic curve (AUC-ROC) have not been reported for any innate immune biomarker specifically in EP cohorts. Without these metrics, optimal cut-off values cannot be established, and the discriminative ability of these markers remains unknown. Second, measurement error and data standardization pose major challenges. Variability in assay protocols (e.g., different ELISA kits), intra- and inter-assay variation, differences in sample collection timing (e.g., time from onset of symptoms), and inter-laboratory calibration differences can significantly affect biomarker reliability. Future studies must adopt standardized operating procedures, report assay validation parameters, and consider calibration models or measurement error correction techniques. Third, the lack of prospective, multi-center validation in diverse populations limits external validity. Most current evidence comes from single-center studies with small sample sizes, often underpowered to detect clinically meaningful effect sizes. We strongly recommend that future research prioritize ROC-based performance evaluation, establish EP-specific cut-offs, and adhere to reporting guidelines such as STARD or REMARK.

## 6 Conclusion

In conclusion, the pivotal and intricate role of innate immunity in the pathogenesis of EP has been increasingly elucidated, underscoring its dual function in pathogen recognition and the initiation of inflammatory cascades, as well as its profound influence on tissue injury and repair mechanisms. This comprehensive understanding bridges the gap between molecular immunology and clinical manifestations, highlighting innate immune components not merely as bystanders but as active drivers in disease progression. Balancing diverse research findings reveal that while innate immunity triggers the early inflammatory response essential for pathogen clearance, its dysregulation significantly contributes to pancreatic tissue damage and chronic disease.

Advances in the identification of innate immune-related biomarkers and the application of advanced diagnostic technologies have markedly improved the early detection and precise assessment of EP severity. These tools provide clinicians with invaluable insights into patients’ immunological status, enabling timely intervention and more accurate prognostication. Moreover, such biomarkers serve as critical prognostic indicators, facilitating patient stratification according to disease severity and expected outcomes. However, heterogeneity in biomarker expression and diagnostic outcomes across different patient populations calls for cautious interpretation and further validation. Harmonizing these diagnostic approaches with clinical parameters is therefore essential to refining EP management protocols. Therapeutically, strategies targeting innate immune modulation hold considerable promise. Immunomodulators and cell-based therapies have demonstrated encouraging efficacy in preclinical and early clinical settings, offering novel avenues to mitigate inflammation while promoting tissue repair. These approaches aim to precisely modulate immune activation, preserving essential host defense mechanisms while curbing excessive inflammation. Nevertheless, the complexity of the innate immune network necessitates a nuanced treatment design to avoid compromising host defenses or exacerbating infection risks. This delicate balance underscores the importance of personalized medicine, where interventions are tailored to individual immunological profiles and disease stages.

Future research must prioritize unraveling the intricate mechanisms governing innate immune responses in EP, employing advanced molecular and systems biology techniques to delineate the interplay between immune cells, signaling pathways, and the pancreatic microenvironment. Innovations in diagnostic methodologies should aim to enhance sensitivity, specificity, and real-time monitoring capabilities, enabling dynamic assessment of disease trajectory. Furthermore, the development of individualized therapeutic regimens, informed by comprehensive immunophenotyping and genetic profiling, is imperative to optimize clinical outcomes and reduce morbidity. Interdisciplinary collaboration integrating immunology, molecular biology, and clinical sciences will be crucial in translating these mechanistic insights into practical, personalized treatment paradigms. In summary, the evolving landscape of innate immunity research in EP provides a robust framework for improving diagnosis, treatment, and prognosis. By harmonizing diverse research perspectives and fostering a balanced interpretation of emerging data, the medical community can develop more effective, tailored strategies to address the challenges of EP. This integrative approach will not only deepen our comprehension of EP pathophysiology but also translate into tangible clinical benefits, ultimately enhancing patient survival and quality of life.

## 7 Limitations

### 7.1 Methodological limitations of this narrative review

The authors acknowledge several methodological limitations of this narrative review. First, no formal risk-of-bias assessment was performed using tools such as ROBINS-I, the Newcastle-Ottawa Scale, or SYRCLE, which is a limitation given the predominance of preclinical and observational studies in the literature. Consequently, the strength of the conclusions should be interpreted as hypothesis-generating rather than definitive. Second, as a narrative review, we did not conduct a quantitative meta-analysis; effect sizes (odds ratios, hazard ratios, confidence intervals) and heterogeneity metrics (I², tau²) are not reported. Third, the review is limited by the available EP-specific literature, which is sparse; many inferences are extrapolated from studies of sterile acute pancreatitis or other infectious conditions. Fourth, study heterogeneity (differences in EP diagnostic criteria, severity scoring systems, timing of biomarker measurement, and outcome definitions) across the included literature may have influenced the consistency of the findings and limits the generalizability of our conclusions.

### 7.2 Inclusion of animal data

Our review included 12 animal studies. Findings from animal models of pancreatitis may not fully translate to human EP pathophysiology due to species differences in immune responses, disease induction methods, and the absence of spontaneous gas-forming infection in most models. Therefore, animal-derived mechanistic insights should be interpreted with caution.

### 7.3 Causality and confounding

A key limitation inherent in synthesizing evidence from predominantly observational and correlative studies is the inability to firmly establish causality. Although mechanistic experiments suggest pathways, clinical associations between biomarker levels and outcomes may be confounded by factors such as disease severity, comorbidities, timing of measurement, and treatment protocols. We have revised the manuscript to replace causal language with associative terms. Future prospective studies employing multivariable regression models to control for potential confounders, as well as more sophisticated causal inference frameworks (e.g., Mendelian randomization, instrumental variable analysis), are needed to move from association to causation.

### 7.4 External validity and generalizability

The generalizability of the findings summarized here may be limited. Most studies are conducted in single centers, specific intensive care settings, or homogeneous populations. Biomarker performance can vary significantly with disease severity, patient age, comorbidities, and the causative pathogen. Furthermore, most cited studies are not powered *a priori* to detect clinically meaningful effect sizes for biomarker performance, and sample size considerations are rarely discussed. These factors must be rigorously addressed in future large-scale, multi-center, prospective validation studies before any of these biomarkers can be reliably integrated into routine clinical practice.

### 7.5 Evidence level classification

To aid the reader, we have classified the evidence underlying key statements in this review:

- High (human EP cohorts with controlled designs): None available to date.
- Moderate (human studies in acute pancreatitis (non-emphysematous) or validated biomarkers in other infectious/inflammatory conditions that share pathophysiological features with EP.
- Low (preclinical models of pancreatitis, e.g., caerulein-, L-arginine-, or taurocholate-induced): Most mechanistic insights into NLRP3, NETosis, macrophage polarization, and MAPK/NF-κB signaling.
- Very low (extrapolation from non-pancreatic diseases or *in vitro* studies only): e.g., PTX3, CLRs, RLRs in EP.

Readers should interpret the review’s conclusions with these evidence levels in mind.

### 7.6 Future perspectives: advanced analytical frameworks

**Figure 2:**
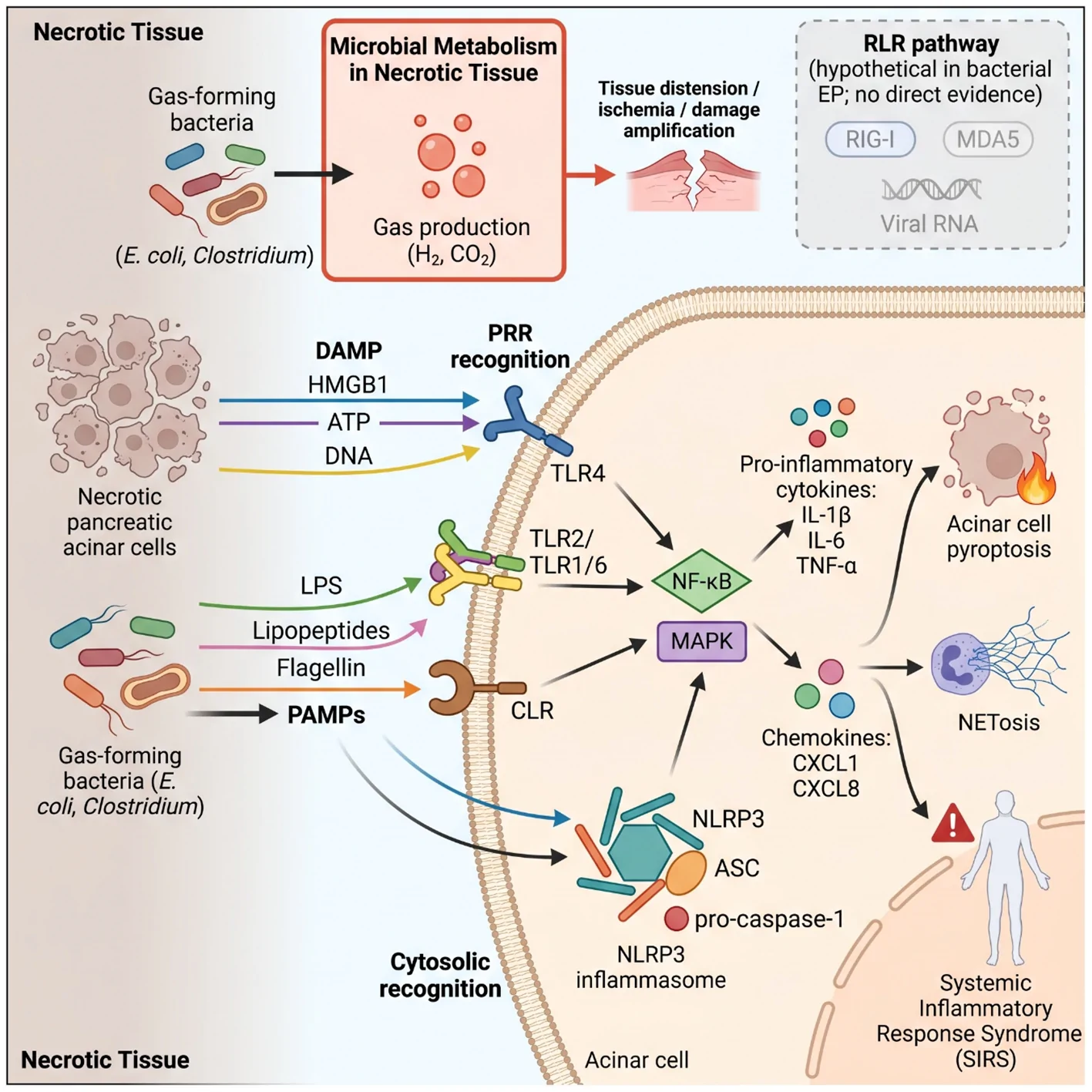
PRR signaling network in EP. TLRs, CLRs, and NLRP3 inflammasome recognize PAMPs/DAMPs and converge on NF-κB/MAPK pathways, producing cytokines (IL-1β, IL-6, TNF-α) and chemokines (CXCL1, CXCL8), leading to pyroptosis, NETosis, and SIRS. H_2_ and CO_2_ are shown as byproducts of microbial metabolism, not as PRR agonists; the RLR-viral RNA pathway is omitted (no evidence in EP).

**Figure 3:**
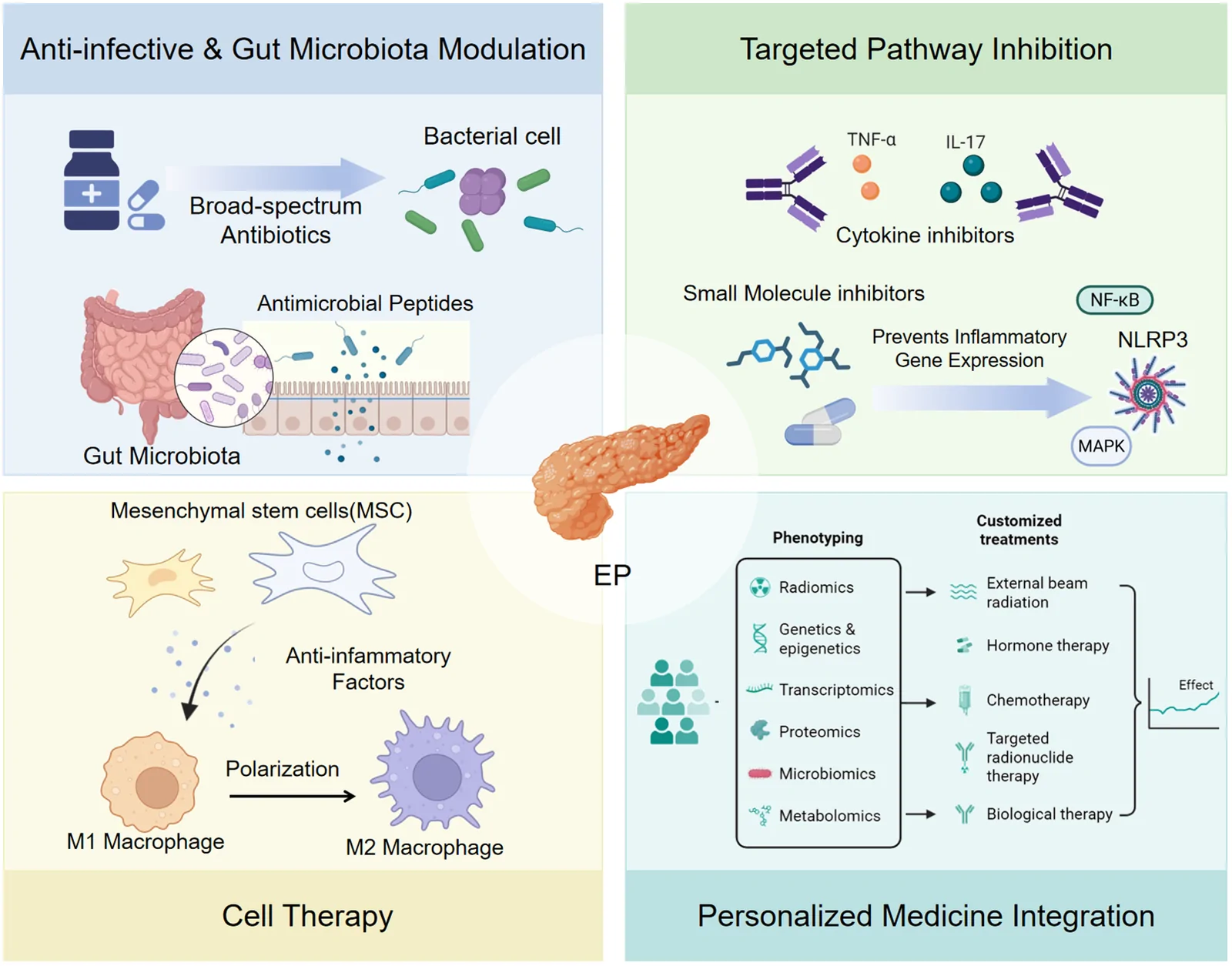
A multimodal therapeutic framework targeting innate immunity for EP(qualitative schematic). This schematic illustrates an integrated, multimodal framework for treating EP by targeting dysregulated innate immunity. The strategy progresses from foundational antimicrobial therapy and immunomodulation to directly inhibit key inflammatory pathways using biologics and small-molecule inhibitors. It further incorporates advanced reparative approaches, such as mesenchymal stem cell therapy and culminates in a precision medicine model where multi-omics profiling guides the optimal selection and combination of these interventions. The overall framework highlights the potential of combining sequential and synergistic strategies to mitigate inflammation, promote tissue repair, and improve clinical outcomes.

To fully harness high-dimensional biological data in EP research, future studies should integrate modern analytical frameworks. Network analysis could map interactions between multiple biomarkers (cytokines, PRRs, metabolites) and identify key regulatory hubs. Machine learning algorithms (e.g., random forests, support vector machines, XGBoost) can integrate multi-omics data (genomics, transcriptomics, proteomics) to improve predictive accuracy for disease severity or prognosis. Longitudinal mixed-effects models are better suited for analyzing dynamic changes in inflammatory mediators over time, capturing within-patient trajectories. Bayesian hierarchical models can handle heterogeneity across small studies and incorporate prior information. Additionally, pathway enrichment analysis of transcriptomic or proteomic data could validate the activity of PRR signaling networks illustrated in **Figure 2**, while network centrality metrics could identify hub molecules as potential therapeutic targets. We encourage researchers to apply these methods in future EP cohorts. Organ-on-a-chip and tissue-on-a-chip technologies may also provide physiologically relevant platforms for modeling EP and evaluating candidate interventions (62). A multimodal therapeutic framework integrating anti-infective, pathway-targeted, cell-based, and precision-medicine approaches is shown in **Figure 3**.
